# THz QCL-Based Cryogen-Free Spectrometer for *in Situ* Trace Gas Sensing

**DOI:** 10.3390/s130303331

**Published:** 2013-03-11

**Authors:** Luigi Consolino, Saverio Bartalini, Harvey E. Beere, David A. Ritchie, Miriam Serena Vitiello, Paolo De Natale

**Affiliations:** 1 CNR, Istituto Nazionale di Ottica and LENS (European Laboratory for Non-linear Spectroscopy), Via Carrara 1, Sesto Fiorentino (FI) 50019, Italy; E-Mails: saverio.bartalini@ino.it (S.B.); miriam.vitiello@sns.it (M.S.V.); paolo.denatale@ino.it (P.D.N.); 2 Cavendish Laboratory, University of Cambridge, J J Thomson Avenue, Cambridge CB3 0HE, UK; E-Mails: heb1000@cam.ac.uk (H.E.B.); dar11@cam.ac.uk (D.A.R.); 3 NEST, CNR, Istituto Nanoscienze and Scuola Normale Superiore, Piazza San Silvestro 12, Pisa 56127, Italy

**Keywords:** terahertz spectroscopy, quantum cascade lasers, trace gas sensing

## Abstract

We report on a set of high-sensitivity terahertz spectroscopy experiments making use of QCLs to detect rotational molecular transitions in the far-infrared. We demonstrate that using a compact and transportable cryogen-free setup, based on a quantum cascade laser in a closed-cycle Stirling cryostat, and pyroelectric detectors, a considerable improvement in sensitivity can be obtained by implementing a wavelength modulation spectroscopy technique. Indeed, we show that the sensitivity of methanol vapour detection can be improved by a factor ≈ 4 with respect to standard direct absorption approaches, offering perspectives for high sensitivity detection of a number of chemical compounds across the far-infrared spectral range.

## 1. Introduction

After three decades from the first applications of phase/frequency modulation techniques to optical spectroscopy with standard laser systems [1], their transfer to other spectral regions such as the terahertz is still to be exploited. More recently, powerful sources such as diode lasers or quantum cascade lasers (QCLs) allowed easier access to the near-infrared (NIR) and mid-infrared (MIR) spectral regions [2,3], also proving to be particularly suitable for these techniques, as they allow direct modulation of the optical radiation by means of a modulation of the supplied current. Nevertheless, the terahertz (THz) region of the electromagnetic spectrum is still largely unexplored, due to limited options of tunable and powerful laser sources, optical components and detectors.

THz molecular spectroscopy has an amazing scientific potential since many absorption and emission molecular lines of interest in astrophysics and atmospheric sciences fall in this spectral region, where many chemical species have very strong characteristic rotational and ro-vibrational transitions [4]. In particular, for gases, the typical absorption strengths are 10^3^–10^6^ stronger than in the microwave region [5]. Moreover, it is estimated that one-half of the total luminosity of the galaxy and 98% of the photons emitted since the Big Bang fall into the terahertz range [6]. Much of this radiation is emitted by cool interstellar dust inside our own and other galaxies, and thus the study of the continuum radiation, as well as of the discrete lines emitted by light molecular species, gives insights into star formation and decay. The terahertz also contains information on the cosmic background, and very distant newly formed galaxies [7].

For atmospheric physics, thermal emission from gases in the stratosphere and upper troposphere such as water, oxygen, chlorine, and nitrogen compounds is useful for the study of chemical processes related to ozone depletion, pollution monitoring, and global warming, as well as direct detection of traces of such gases in open air [8,9]. Other spectroscopic applications include plasma fusion diagnostics, where temperature profiles of the plasma can be obtained [10] or, for safety and security applications, spectroscopic identification of different crystalline polymorphic states of drugs.

Laboratory spectroscopic techniques with a resolution of Δ&upsilon;/&upsilon; ≈ 10^−6^ at THz frequencies are a powerful tool for investigation of the structure and energy levels of molecules. Besides analysis on the nature of the specific species, important information on lineshape broadening and frequency shifts due to varying external parameters can be obtained from THz spectra. Sensitive, high-resolution spectroscopy has been successfully performed in the sub-THz range without the need for an illuminating source or, alternatively, by detecting tunable narrowband terahertz radiation after transmission or reflection through some medium of interest. However, despite the wide variety of methods successfully developed at frequencies ν ≤ 1 THz, spectroscopy at frequencies ν >1–2 THz has been hindered by the lack of frequency tunable and narrow linewidth radiation sources.

To address the requirements for THz spectroscopic applications, several technologies providing tunable THz emission have been developed in the last few decades. One possible approach is to combine the radiation of two CO_2_ lasers in a metal-insulator-metal diode which generates a difference frequency in the THz range [11–13]. By proper choices of laser lines, this approach demonstrated an overall tunability range from the microwave region up to about 9 THz, with a continuous tunability, for fixed laser lines, of ≈40 GHz, and a few hundred nanowatts of power [14]. Another approach was based on mixing the radiation of an optically pumped THz gas laser with the radiation of a microwave source or a backward wave oscillator (BWO) in a Schottky barrier diode [15]. By this method, sidebands are generated whose frequency can be tuned by changing the frequency of the BWO or microwave source. These systems typically generated only several μW of output power and suffered from complexity. In spite of these limitations, a relevant number of physical results on molecules and atoms could be achieved [16–19]. In the second approach, frequency coverage was also limited, because only a limited number of powerful gas laser lines are available for sideband generation. The sensitivity of any spectroscopic experiment relying on one of these techniques was ultimately limited by the available power. Terahertz QCLs are a promising alternative which might significantly improve sensitivity.

Since their first demonstration [20], the performance of THz quantum cascade lasers improved dramatically in terms of output power, operation temperature, frequency tunability [21,22], and stability, making QCL technology the more attractive choice across the far infrared. THz QCLs have been used, in the last few years, for imaging and high resolution molecular spectroscopy, taking advantage of their inherent coherence and quantum limited linewidth [23]. In addition, application of QCLs as frequency and amplitude stabilized local oscillators (LO) in THz heterodyne spectrometers has been only recently explored [24]. In addition, THz QCLs have recently shown a quantum limited linewidth of ≈100 Hz. This means that, in principle, once properly frequency stabilized, QCLs can be used for ultra-high resolution molecular spectroscopy, paving the way to a number of key applications as well as to fundamental studies on molecules. Nevertheless, for many spectroscopic applications of practical interest, the crucial parameter is sensitivity, while resolution requirements are often not so challenging. This is, for example, the case of atmospheric trace gas detection, where typical transition linewidths can be broadened up to a few tens of GHz, or of spectroscopy in a low-pressure gas, where Doppler broadening, at THz frequencies and room-temperature, is in the range of a few MHz for many of the most common molecules. Actually, this is the case studied in this article, where ro-vibrational molecular transition lines of CH_3_OH gas, around &upsilon; ≈ 2.5 THz, are detected at low pressure, with a Doppler limited linewidth of about 5.5 MHz at 300 K.

The latest studies on THz spectroscopy based on QCLs make use of direct absorption and wavelength modulation spectroscopy [25–27]. Other approaches are based on coupling a QCL to a THz mixer in a heterodyne configuration with a hot electron bolometer (HEB) [28] or a Schottky diode [29].

In this paper, we report on a set of spectroscopy experiments using QCLs to detect rotational molecular transitions in the far-infrared spectral region, based on a differential spectroscopy approach, aimed to improve the detection sensitivity. We demonstrate that with our room temperature, compact and transportable setup, based on a closed-cycle Stirling cryostat and pyroelectric detectors, an improvement in sensitivity can be obtained by implementing a wavelength modulation spectroscopy (WMS) technique, with a differential detection scheme.

## 2. Terahertz Spectroscopy with QCLs

Our spectrometer has been characterized at 118 micron wavelength by spectroscopy on methanol gas, but it can work at different wavelengths by changing the QCL source.

WMS is a derivative form of absorption spectroscopy that has been increasingly applied for measurements in harsh environments, due to its improved sensitivity and noise-rejection capabilities, as compared to direct absorption. In the WMS technique the laser wavelength is modulated with a sinusoid (at a frequency *f*) and the harmonic components in the detector signal are conventionally singled out with a lock-in amplifier. The modulation frequency *f* is lower than the optical frequency half-width of the probed absorption feature (kHz to several MHz range).

The QCL used for this work is based on a bound-to-continuum (BTC) design. It has been then soldered to a copper bar, wire bonded, and mounted on the cold finger of a mechanical closed-cycle cryo-cooler (Ricor, mod. K535). In order to minimize the influence of mechanical vibrations of the cryo-cooler, flexible copper wires have been used for connecting the laser to the cold finger of the cooler. The cooler has a heat extraction capacity of ≈1 W at 4 K. The laser was driven in continuous wave mode at a fixed heat sink temperature of ≈47.5 K. Under this experimental condition, the threshold current density was J_th_ ≈ 100 A/cm^2^ (*i.e.*, I_th_ = 375 mA), with a maximum output power P_out_ = 2.5 mW. The QCL frequency can be tuned by either changing its operation temperature or by applying a sweep current reaching a maximum tunability range of a few GHz. The tuning-by-current coefficient (≈8.5 ± 0.5 MHz/mA) of the QCL has been already measured in [23], and has been confirmed in an independent experiment, where the QCL frequency was measured with respect to an absolute frequency reference [30].

**Figure 1.**
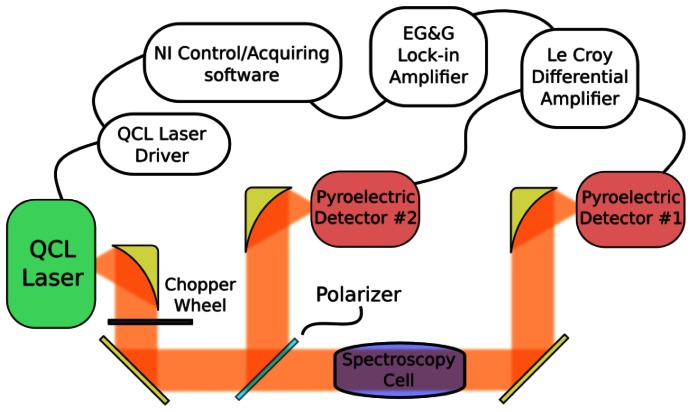
Experiment apparatus used for high sensitivity spectroscopy. The THz QCL laser is housed in a closed-cycle Stirling Cryostat, so that it can be operated at room temperature. Therefore the whole set-up has been mounted on a compact and transportable breadboard, allowing *in situ* measurements (spectrometer overall dimensions: 90 × 60 × 90 cm, total weight: <100 kg).

A schematic draft of the experimental set-up is shown in Figure 1. In comparison with the direct absorption scheme, the differential acquisition of two signals permits singling out the specific contribution due to the molecular transition in the gas sample from any other external fluctuation, such as the background slope due to the scanning laser current or laser temperature instability. The continuous wave (CW) THz radiation coming from the QCL is chopped at a fixed frequency of 130 Hz, and it is collected and collimated by a 90° off-axis parabolic mirror. The beam is then split into two parts by means of a wire grid polarizer. The first one, used to provide the power reference, is directly focused onto a pyroeletric detector (P2). The second part is sent through the spectroscopy cell (20 cm length, 1 inch internal diameter, with 2 mm thick TPX windows) filled with methanol vapor at the desired pressure, positioned along the beam path. Light is then focused by means of a parabolic mirror onto another, identical, pyroelectric detector (P1). The two signals are balanced onto a differential amplifier (mod. DA1822A, Teledyne LeCroy, Chestnut Ridge, NY, USA), and the difference is then demodulated by a lock-in amplifier (mod. 5209, Eg&G, San Francisco, CA, USA). P1 and P2 are two identical pyroelectric detectors (mod. QS2-THZ-BL, Gentec-EO, Quebec, Canada), with a NEP of 4.0 × 10^−1^° W/Hz^−1/2^, a responsivity of about 140 kV/W, and a −3 dB bandwidth of 30 Hz. The 130 Hz modulation frequency provides not only a faster acquisition, but also a 15 dB attenuation that reduces the output signal down to few Volts. Digital input-output electronics, controlled by a personal computer equipped with a self-programmed LabView software, acquires the spectroscopy signal and controls the QCL parameters, such as operating temperature and current. In this way all the control and acquisition tasks are embedded in the same unit.

## 3. Results and Discussion

**Figure 2.**
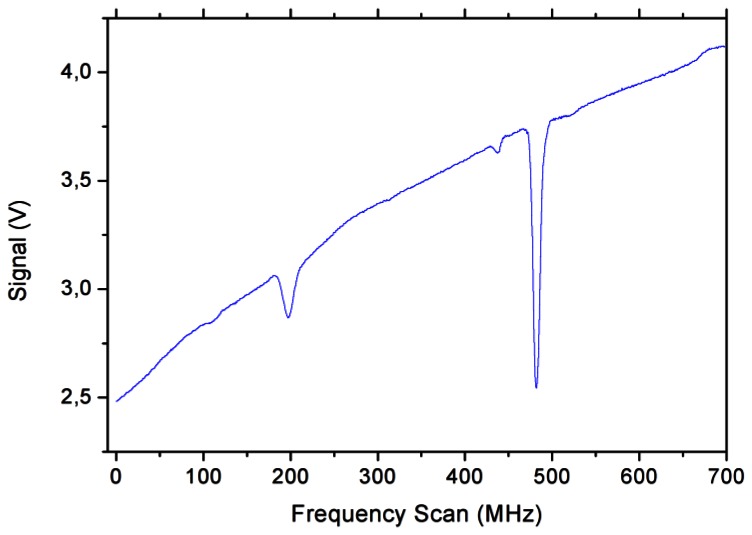
Absorption profile of methanol vapor at a pressure P = 0.4 mbar, obtained by direct spectroscopy. The laser frequency is scanned by changing the driving current. The measured width of the most intense line is about 8 MHz (FWHM).

Figure 2 shows the absorption profile acquired with a conventional direct spectroscopy scheme, obtained by stopping the reference beam incident on P2. The laser frequency is swept by changing the driving current, and consequently the covered portion of the molecular spectrum is acquired. As expected, the molecular absorption signal is affected by the strong dependence of the QCL power on the sweeping current. This usually requires a post-processing of the data, in order to remove the background and to properly fit the absorption profiles. The signal-to-noise (S/N) ratio achieved is about 270:1.

**Figure 3.**
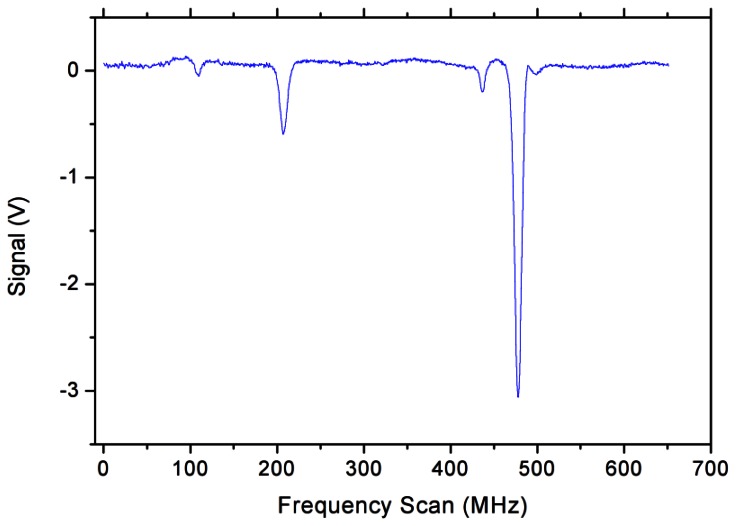
Differential acquisition spectroscopy of the absorption profile. The background slope of Figure 2 is automatically canceled out, since only deviations of the signal from the reference beam are actually detected. The measured width of the most intense line is about 8 MHz (FWHM).

In the case of the differential acquisition, the acquired data have no background slope, as the latter is automatically cancelled in the acquisition procedure. Therefore, the signal can be eventually fitted with a multi-peak function in order to retrieve the intensity of the absorption lines and their positions. A typical frequency scan is shown in Figure 3. In terms of S/N ratio, the differential acquisition provides a slight improvement: the measured S/N ratio is 490:1, *i.e.*, 45% more than that obtained with the direct absorption scheme.

**Figure 4.**
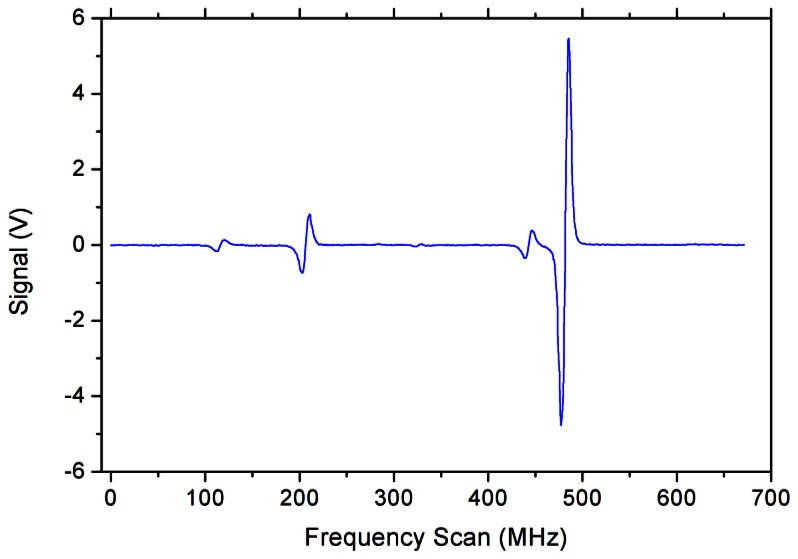
WMS acquisition of the absorption profile, at the same methanol vapor pressure of 0.4 mbar, taken with a modulation amplitude of 10 mV_pp_. The measured width of the most intense line is about 9 MHz (FWHM).

To further increase the sensitivity of our spectrometer, we implemented a WMS technique. It further simplified the experimental setup, since the chopper is no more required, as the 130 Hz modulation is sent to the laser through the driving current. The resulting frequency modulation leads to a detectable amplitude modulation only when in resonance with a molecular line. We used a bias-tee on the analog control input of the current driver in order to sum the slowly scanning laser current together with a small modulation (few per cents relative amplitude). The differential signal is then demodulated by a lock-in amplifier, obtaining the first derivative of the absorption profiles. The complete WMS scan of the same set of lines shown in the previous figures is reported in Figure 4.

**Figure 5.**
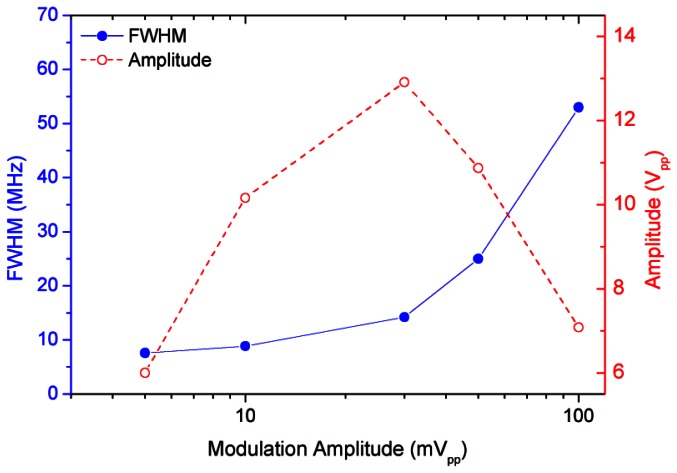
Dependence of the amplitude and width of the WMS signal on the amplitude of the modulation signal. A modulation of 10 mV_pp_ ensures a larger signal without any significant profile deformation.

The S/N ratio of the WMS signal can be optimized by increasing the amplitude of the modulating signal. From Figure 5, however, it can be noticed that too high values of the modulation amplitude results in degradation of the WMS signal. Therefore, the chosen modulation amplitude was fixed to 10 mV_pp_, corresponding to the maximum signal detectable without any significant deformation.

In the optimized conditions, the S/N reached by the WMS technique is 1,250:1, more than a factor of 4 larger than using direct absorption spectroscopy. The ultimate sensitivity achievable with our spectrometer can be thus identified with the minimum detectable absorption coefficient (α_min_). From the comparison between the S/N ratios of direct absorption and WMS trace, we conclude that an α_min_ ∼ 5.5 × 10^−6^ cm^−1^ Hz^−1/2^ is achievable. Other state-of-art spectrometers, based on a multiplied microwave source [31] or on a QCL [26], achieve slightly higher sensitivities (2.5 × 10^−6^ cm^−1^ Hz^−1/2^ and approximately 10^−6^ cm^−1^ Hz^−1/2^), mostly thanks to the use of sophisticated cryogenic detectors (a composite silicon bolometer and a Ge:Ga photoconductive detector, respectively).

The application of our spectrometer to detection of traces of hydrogen sulfide (H_2_S) in atmosphere is under study. Hydrogen sulfide is a highly toxic and flammable gas, heavier than air. The concentration limits set by the US. Occupational Safety and Health Administration (OSHA) are 20 parts per million (ppm) for long lasting exposure, and a peak limit of 50 ppm for no longer than 10 min, if no other measurable exposure occurs [32]. Inhalation of concentrations of 500–1,000 ppm will cause rapid unconsciousness and death through respiratory paralysis and asphyxiation. Although very pungent at first, it quickly deadens the sense of smell, so potential victims may be unaware of its presence until it is too late. A set of three absorption lines of H_2_S molecule was selected around 108.5 cm^−1^, falling right in the middle of a transparency window of water vapor. This is a stringent requirement for any high-sensitivity detection in atmosphere, where strong absorption of water vapor can easily suppress the signal. From the above presented results, we expect that, by replacing the actual 20-cm-long cell with a 1-m-long cell, the ultimate detectable concentration of H_2_S in dry air would be about 200 ppb, that is 1% of the acceptable threshold concentration

## 4. Conclusions

THz molecular spectroscopy has a remarkable scientific potential since many absorption and emission molecular lines of interest in astrophysics and atmospheric sciences fall in this spectral region, where many chemical species have rotational and ro-vibrational transitions 10^3^–10^6^ stronger than in the microwave region. Using a 2.5 THz quantum cascade laser we performed high sensitivity spectroscopy experiments in both direct, differential, and WMS configurations. Our results show that WMS in a differential scheme is a very powerful tool with the potential to achieve sensitivity levels down to hundreds of ppb for the detection of molecules, drugs, hazardous chemicals and gases having a strategic relevance across the Terahertz spectral range. Moreover, the possibility to implement a compact THz system based on a portable closed-cycle Stirling cryo-cooler and a room-temperature pyroelectric detector paves the way to a full solid-state, either *in-situ* or on open air, photonic platform relevant for strategic fields as security, astronomy, environmental monitoring, and cultural heritage.
